# Factors Predisposing to the Onset of Bipolar Disorder: A 30‐Year Longitudinal Study

**DOI:** 10.1111/bdi.70026

**Published:** 2025-03-28

**Authors:** Peter Tyrer, Min Yang, Helen Tyrer, Gin Malhi

**Affiliations:** ^1^ Division of Psychiatry Imperial College London England; ^2^ School of Social Sciences Nottingham Trent University Nottingham England; ^3^ Swinburne University of Technology Melbourne Victoria Australia; ^4^ West China School of Public Health Sichuan University Chengdu China; ^5^ Faculty of Medicine & Health University of Sydney Sydney Australia

**Keywords:** bipolar disorder, long‐term outcome, mixed anxiety‐depression, prediction

## Abstract

**Objective:**

To examine the factors that predict the development of bipolar disorder in a population presenting with anxiety and depressive disorders.

**Method:**

In a 30‐year study, the Nottingham Study of Neurotic Disorder, the personality status, life events, service data, and early course of patients recruited to a randomised controlled trial were compared in patients who developed bipolar pathology and those who had no bipolar symptoms.

**Results:**

Over 30 years, 5 (2.5%) of 200 patients assessed at baseline developed unequivocal bipolar disorder, one within the first 10 weeks of the study, and three (1.5%) had bipolar II pathology. Analysis of these data showed that those patients who had some degree of bipolarity had an increase in anxiety and depressive symptoms and general psychopathology, most pronounced in the second year of the study, that was not found with patients who had no bipolar pathology.

**Conclusions:**

Patients treated for anxiety and depressive disorders who remain unwell after initial treatment are more at risk of developing bipolar disorder than others.

## 1 Introduction

It is well established that the course of bipolar disorder may be preceded by other affective symptoms, and these may precede the first episode of hypomania or mania by many years. Apart from genetic predisposition and childhood instability of mood, there are few data examining affective symptoms and personality status in those that develop bipolar disorder.

This has led to debate about identifying the precursors and predicting the onset of bipolar disorder, an issue that might be relevant to prevention. Some researchers claim it is a useless exercise that can only be explained by chaos [1], while others hope that advances in neurobiology, including the combination of genetic predisposition and instability of mood in adolescence, will enable earlier and more effective treatment [2, 3].

The factors formulated as precursors of bipolar disorder are all at a low level of confidence. They include (a) a preceding period of dysthymia [4], (b) strong extraversion characteristics on personality measures [5], (c) unresolved anxiety [6] and (d) high levels of substance misuse at baseline [7].

In a 30‐year study, the Nottingham Study of Neurotic Disorder, which started collecting data in 1983, clinical, social function, service changes, GP records, life events, and personality status were recorded at regular intervals over the 30‐year period, with most interviews (nine) carried out in the first 2 years of the study.

As some of the patients included in the study developed bipolar pathology, the data base offered an opportunity to examine the factors that might predispose to bipolarity and so was examined in detail.

## 2 Method

Patients in the Nottingham Study of Neurotic Disorder were recruited over a four‐year period between 1983 and 1987. Those recruited all had diagnoses of anxiety and depressive disorders and were all seen in general practice psychiatric clinics. These clinics were popular extensions of community care in the latter years of the 20th century [8, 9] and saw patients at an earlier stage in their pathology [10]. Because the referred patients were seen early, they were also more representative of the population with anxiety and depressive disorders, who at the time of the study were classified as the core group of neurotic disorders [11].

The patients took part in a randomised trial of treatments in the first 10 weeks of the study, the results of which have been published elsewhere [12]. They had further assessments at 16, 32, and 52 weeks, and after 2, 5, 12 and 30 years after randomisation, with the last patient being seen in July 2019. Inclusion criteria were (i) no active psychiatric treatment at entry, (ii) informed consent, (iii) a Diagnostic and Statistical Manual of Mental Disorders (DSM‐III) diagnosis [13] of either GAD, panic disorder, or dysthymic disorder (or any mixture of these), determined by the administration of the Structured Clinical Interview for DSM‐III [14] and (iv) no history of other assumed independent psychiatric illness (schizophrenia, bipolar disorder or alcohol or drug addiction). Two hundred and ten patients were randomised, but 10 patients dropped out or were found to be ineligible before baseline measures were completed.

### 2.1 Assessments

The aims of the study included measurement of symptomatic and personality change [12, 15, 16], recording of life events and environmental change [17, 18], changes in the SCID DSM diagnosis [14], and treatment and service use data over the follow‐up period [19, 20, 21].

Symptomatic change was recorded by interview using the Comprehensive Psychopathological Rating Scale [22], and two related subscales, the Montgomery & Åsberg Depression Rating Scale (MADRS) [23] and the Brief Scale for Anxiety (BAS) [24]. Self‐ratings of anxiety and depression were recorded using the Hospital Anxiety and Depression Rating Scale (HADS) [25]. Because many patients had significant anxiety and depressive symptoms, the scores on the HADS scale were combined and classed as cothymia [26, 27].

Life events up to 2 years were recorded using the Scaling of Life Events Scale [28] and subsequently by interview and assessment of clinical notes [19, 21]. Personality assessment was carried out using the Personality Assessment Schedule (PAS) [29, 30] and its subsequent conversion to DSM‐III categories of personality disorder [31].

Assessments in the study took place between 1983 and 2019. As the patients were recruited over a four‐year period, the follow‐up times were also staggered. The study began as a randomised trial of five treatments: placebo, dothiepin, diazepam, cognitive behaviour therapy and self‐help, given for 6 weeks and then tailed off before assessment at 10 weeks [12]. Personality assessment was also made at baseline. Subsequently, further assessments were made at 16, 32, 52 and 104 weeks after randomisation, when further data were reported [32]. After 5 years, assessments were made by examination of case notes only and at 12 and 30 years by face‐to‐face interviews. After 30 years, the Hypomania Checklist (HCL‐32) [33] was administered to all interviewees.

## 3 Results

### 3.1 Patients With Bipolar Symptoms

Twelve of the 200 patients showed some evidence of bipolar symptoms over the course of the 30 years but only five developed unequivocal bipolar disorder. These five patients also experienced a total of five further episodes in the 30 years. The first episode was noted 9 weeks after randomisation and was at least partly attributed to cognitive therapy [34], and the last first appearing at the end of the 30‐year period (Table 1). All these episodes led to in‐patient treatment and four of the patients received treatment with lithium salts.

**TABLE 1:** Five patients with unequivocal bipolar symptoms.

Study ID	Age at trial entry and gender	Time after randomisation of first episode of bipolar disorder	Number of subsequent episodes	HCL score (when recorded at 30 years)
10	F	9 weeks	2	Died before follow‐up
172	M	27 months	1	Died before follow‐up
03	M	28 years	3	16
42	F	21 years	0	23
201	F	24 years	0	Lost to follow‐up

*Note:* ID10 F Hypomanic episodes at 5 weeks and 10 years (admitted to hospital each time). ID172 M Manic episodes 27 m and 32 m after randomisation (admitted to hospital) died Oct 89 from gastric carcinoma. ID003 M First manic episode Nov. 2012, admitted to hospital. Diagnosis of bipolar disorder first made at this time. Further episodes in Feb/Mar 2013, Feb/Mar 2015 and July 2015. Had previous psychiatric admission for temazepam dependence and withdrawal in 1992 HCL score at 20 years 16. ID42 F First manic episode Sept 2005. Admitted to hospital. Put on lithium, well ever since HCL score at 30 years 23. ID201 F First manic episode in 2011. On lithium ever since.

### 3.2 Patients With Possible Bipolar II Disorder

Two of the patients had periods of depression and anxiety over the first 12 years and also had a single hypomanic episode of 12–20 days. These were not diagnosed as bipolar disorder but in the new ICD‐11 classification would qualify for Bipolar type II disorder [35, 36].

### 3.3 Patients With Elevated HCL Scores at 30 Years

Five further patients had HCL‐32 scores of 15 or more at 30‐year follow‐up. There were no clear episodes of hypomania in their histories and considerable overlap with periods of emotional instability. However, because in some cases apparent episodes had occurred up to 15 years previously and HCL‐32 scores were still raised, these were classed as possible Bipolar II disorder in the analyses. The HCL‐32 scores do not allow a distinction to be made between Bipolar I and II disorder [33].

### 3.4 Comparison of Course of Disorder

The further analyses examined the three groups of possible bipolarity separately.

### 3.5 Life Events

No differences were found between total life event scores or events separated by loss, arrival, or conflict (Table 2).

**TABLE 2:** Life events separated by bipolar disorder (BD) groups.

BD group	10 weeks	52 weeks	2 years
**Event score**	***N*: mean ± SD (95% CI)**	***N*: mean ± SD (95% CI)**	***N*: mean ± SD (95% CI)**
No BD	182: 16.0 ± 15.1 (14.9–17.1)	165: 21.9 ± 19.2 (20.4–23.4)	155: 35.4 ± 27.7 (33.2–37.6)
Definite BD	5: 13.1 ± 11.3 (8.1–18.2)	5: 3.5 ± 5.3 (1.1–5.9)	5: 20.9 ± 16.4 (13.6–28.2)
BD II	2: 20.1 ± 28.4 (0.0–40.1)	2: 17.7 ± 10.3 (10.4–25.0)	1: 7.7 (N/A)
Possible BD	5: 28.5 ± 20.8 (19.2–37.8)	4: 38.5 ± 22.7 (27.2–49.9)	4: 47.5 ± 36.7 (29.2–65.9)
**No. Loss event**	***N*: Median (Min.−Max.)**	***N*: Median (Min.−Max.)**	***N*: Median (Min.−Max.)**
No BD	182: 0 (0–2)	165: 0 (0–3)	155: 1 (0–4)
Definite BD	5: 0 (0–1)	5: 0 (0–0)	5: 0 (0–2)
BD II	2: 0.5 (0–1)	2: 0 (0–0)	1: 0 (N/A)
Possible BD	5: 1 (0–1)	4: 0.5 (0–2)	4: 0.5 (0–2)
**No. arrival event**	***N*: Median (Min.−Max.)**	***N*: Median (Min.−Max.)**	***N*: Median (Min.−Max.)**
No BD	182: 1 (0–3)	165: 1 (0–3)	155: 2 (0.9)
Definite BD	5: 1 (0–2)	5: 0 (0–1)	5: 1 (1–2)
BD II	2: 0 (0–0)	2: 0.5 (0–1)	1: 1 (N/A)
Possible BD	5: 2 (0–2)	4: 1.5 (0–3)	4: 4.5 (0–6)
**No. conflict event**	***N*: Median (Min.−Max.)**	***N*: Median (Min.−Max.)**	***N*: Median (Min.−Max.)**
No BD	182: 0 (0–4)	165: 0 (0–4)	155: 0 (0–5)
Definite BD	5: 0 (0–1)	5: 0 (0–1)	5: 0 (0–1)
BD II	2: 1 (0–2)	2: 1 (0–2)	1: 0 (N/A)
Possible BD	5: 1 (0—2)	4: 1.5 (0–3)	4: 0.5 (0–3)

*Note:* No statistically significant differences were found between the groups at any time for any type of event.

Abbreviations: BD = bipolar disorder, BDII = likely bipolar II disorder.

### 3.6 Personality Status

No important differences were found in the initial assessment of personality status, whether measured by the four group system generated by the Personality Assessment Schedule [29] or the subsequent DSM‐III conversion (Table 3).

**TABLE 3:** Personality status at baseline (PAS and DSM‐III categories).

Classification group (first 4 derived from PAS (29)	No BD *N* = 186–192	BD definite *N* = 5	BD II *N* = 3	Possible BD *N* = 6
Sociopathic: Mean (SD)	1.09 (1.09)	1.36 (1.08)	1.27 (1.10)	2.27 (1.54)
Passive‐dependent: Mean (SD)	1.28 (1.05)	1.32 (0.78)	0.53 (0.23)	1.93 (0.76)
Anankastic: Mean (SD)	1.24 (0.85)	2.04 (1.01)	0.67 (0.64)	1.47 (1.16)
Schizoid: Mean (SD)	0.79 (0.83)	0.88 (0.99)	0.40 (0.53)	1.40 (0.93)
Any DSM‐III diagnoses: *n* (%)	69 (37.1)	3 (60.0)	0 (0)	6 (100.0)
Paranoid: *n* (%)	23 (12.4)	1 (20.0)	0 (0)	2 (33.3)
Schizoid: *n* (%)	5 (2.1)	0 (0)	0 (0)	1 (16.7)
Schizotypal: *n* (%)	9 (4.8)	0 (0)	0 (0)	0 (0)
Any Cluster A: *n* (%)	25 (13.4)	1 (20.0)	0 (0)	2 (33.3)
Histrionic: *n* (%)	24 (12.9)	0 (0)	0 (0)	3 (50.0)
Antisocial: *n* (%)	20 (10.8)	1 (20.0)	0 (0)	2 (33.3)
Borderline: *n* (%)	20 (10.8)	1 (20.0)	0 (0)	1 (16.7)
Narcissistic: *n* (%)	13 (7.0)	0 (0)	0 (0)	0 (0)
Any Cluster B: *n* (%)	38 (20.4)	2 (40.0)	0 (0)	4 (66.7)
Avoidant: *n* (%)	21 (11.3)	0 (0)	0 (0)	0 (0)
Dependent: *n* (%)	20 (10.8)	0 (0)	0 (0)	1 (16.7)
Obsessive‐compulsive: *n* (%)	13 (7.0)	1 (20.)	0 (0)	2 (33.3)
Any Cluster C: *n* (%)	39 (21.0)	1 (20)	0 (0)	3 (5
Passive‐aggressive *n* (%)	15 (8.1)	0 (0)	0 (0)	0 (0)

Abbreviations: BD = bipolar disorder; BDII = bipolar II disorder.

### 3.7 Clinical Symptomatology

**FIGURE 1:**
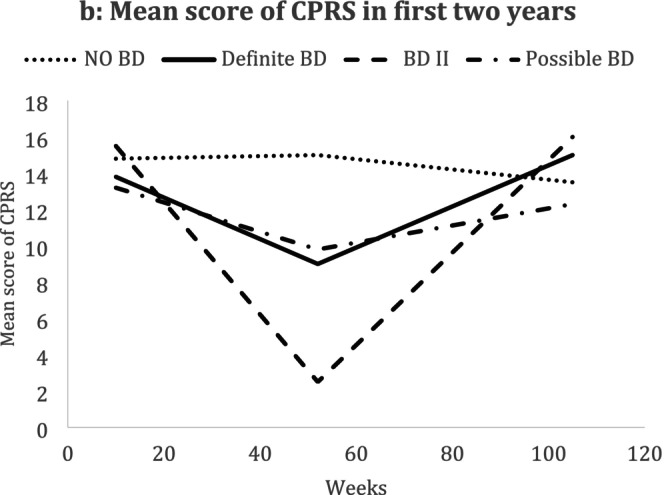
Mean change in scores on the Comprehensive Psychopathological Rating Scale (CPRS) over 2 years, separated by bipolar status.

The only important differences between the groups were found in clinical symptomatology. Total psychopathology, as scored by the Comprehensive Psychopathological Rating Scale (CPRS) [22], in the bipolarity group showed worsening of symptoms over the 2 years, most marked in the second year (Figure 1 and Table 4), and the same trend was shown for those with mixed anxiety and depressive symptoms (identified by total scores on the Hospital Anxiety and Depression Scale (cothymia) (HADS)) (Table 4). The same trend was also shown for depressive symptoms recorded with the MADRS scale (Table 5).

**TABLE 4:** Mean scores on the Comprehensive Psychopathological Rating Scale (CPRS_ from 10 weeks to 2 years separated by subsequent bipolar pathology.

BD group	10 weeks	52 weeks	2 years
	N: mean ± SD (95% CI)	N: mean ± SD (95% CI)	N: mean ± SD (95% CI)
No BD	182: 17.9 ± 9.9 (17.2–18.6)	165: 15.8 ± 9.6 (15.0–16.5)	155: 14.0 ± 9.4 (13.2–14.7)
Definite BD	5: 13.0 ± 12.6 (7.4–18.6)	5: 12.2 ± 14.1 (5.9–18.5)	5: 17.4 ± 15.9 (10.3–24.5)
BD II	2: 14.0 ± 7.1 (9.0–19.0)	2: 5.5 ± 6.9 (1–10)	1: 10.0 (N/A)
Possible BD	5: 18.0 ± 10.3 (13.4–22.6)	4: 10.5 ± 5.7 (7.7–13.4)	4: 15.3 ± 8.8 (10.9–19.7)
F (*p* value)	0.501 (0.682)	1.305 (0.275)	0.289 (0.833)

**TABLE 5:** Mean changes in the depression scores (MADRS scale) [23] of patients separated by bipolarity groups.

BD group	10 weeks	52 weeks	2 years
	N: mean ± SD (95% CI)	N: mean ± SD (95% CI)	N: mean ± SD (95% CI)
No BD	182: 12.8 ± 9.1 (11.6–12.9)	166: 12.0 ± 10.5 (11.2–12.8)	154: 10.5 ± 9.5 (9.7–11.3)
Definite BD	5: 6.2 ± 6.2 (3.4–9.0)	5: 9.0 ± 8.8 (5.1–12.9)	5: 12.6 ± 12.1 (7.2–18.0)
BD II	2: 9.0 ± 12.7 (0.02–18.0)	2: 2.0 ± 2.8 (0.02–4.0)	1: 8.0 (N/A)
Possible BD	5: 12.4 ± 9.9 (8.0–16.8)	4: 11.7 ± 10.3 (6.6–16.9)	4: 10.6 ± 9.4 (5.9–15.3)
F (*p* value)	0.813 (0.488)	0.895 (0.445)	0.111 (0.954)

In the further examination of changes in the CPRS, MADRS, and other measures over time among the bipolar groups and comparing these with the patients with no bipolar, we used two‐level models [37] for repeated measures with adjustment for age, sex and general neurotic symptom status [38] and baseline values. There was a significant increase in the slope of HADS (cothymic group) and MADRS over time of the BD group, although declining trends over time in these measures were found for all patients (Table 6). The increased CPRS score over time for the BD group, as shown in Figure 1 was not significant after adjusting for other factors.

**TABLE 6:** Final model showing the estimated change in slope over time from 10 to 104 weeks in the Nottingham Study.

Clinical and life event measures	Overall change in time		Change in time for the definite BD group	
	Estimated slope (SE)	*p*	Estimated slope (SE)	*p*
HADS (cothymia)	−0.045 (0.007)	< 0.0001	0.093 (0.042)	0.027
CPRS	−0.017 (0.008)	0.033	0.033 (0.045)	0.464
MADRS	−0.020 (0.007)	0.004	0.087 (0.043)	0.043
Life event score	0.205 (0.023)	< 0.0001	−0.113 (0.131)	0.391

*Note:* NB. The results in this table indicated that overall scores in cothymia, CPRS and MADRS declined over time in the non‐bipolar group significantly, but those in the bipolarity BD group increased for cothymia (*p* = 0.027) and MADRS scores (*p* = 0.043). The increase in CPRS did not reach significance. Life event score was significantly increased overall but no difference between the BD and none BD groups. The estimated model supported the observations in the tables and figure above.

## 4 Discussion

As only five patients had unequivocal bipolar disorder over the 30‐year period of follow‐up, the data are insufficient to make any definitive conclusions about predictors of bipolar disorder. Nonetheless, it is reasonable to presume that such a population is more prone to developing bipolar disorder than those with no mental illness. The incidence of affective psychotic disorders has been estimated as 4.6 per 100,000 person years [39]. Our population only comprised 6000 person years, and so less than one person would have been predicted to have an affective psychosis over 30 years if our population was typical of the community.

The consistent finding was that those who had persistent depressive symptoms as well as other psychopathology in the 2 years after initial treatment were likely to develop bipolar pathology subsequently. Even though such pathology might be many years in the future, this precursor is not immaterial. In a staging study in those who had already had at least one episode of bipolar disorder, 75% of patients experienced depressive symptoms before the return of a bipolar episode [40], and it is well established that antidepressants may not be appropriate treatment in these circumstances [4].

## Author Contributions

P.T. was the main writer of the paper. M.Y. carried out the statistical analyses, H.T. carried out the assessments leading to the identification of bipolar pathology, and G.M. helped to improve the balance of the paper.

## Ethics Statement

The initial study was approved by the Nottingham Ethics Committee and subsequently by the Northampton Research Ethics Committee (12/EM/0331).

## Conflicts of Interest

The authors declare no conflicts of interest.

## Data Availability

Data sharing is not applicable to this article as no new data were created or analyzed in this study.
